# Effects on childhood body habitus of feeding large volumes of cow or formula milk compared with breastfeeding in the latter part of infancy^1^,^2^

**DOI:** 10.3945/ajcn.114.100529

**Published:** 2015-09-09

**Authors:** David Hopkins, Colin D Steer, Kate Northstone, Pauline M Emmett

**Affiliations:** 3 Nutrition and Dietetic Department, Southampton General Hospital, Southampton, United Kingdom; and; 4 Centre for Child and Adolescent Health, School of Social and Community Medicine and; 5 School of Social and Community Medicine, University of Bristol, Bristol, United Kingdom

**Keywords:** ALSPAC, BMI, cow milk, formula, growth, height, obesity, weight

## Abstract

**Background: **There is controversy over whether a lack of breastfeeding is related to obesity development.

**Objective: **We examined the effects of feeding different types of milk in late infancy on childhood growth.

**Design:** A cohort of 1112 term, singleton children (born in 1992) from the Avon Longitudinal Study of Parents and Children, United Kingdom, were studied prospectively. Food records collected at 8 mo of age were used to define the following 5 mutually exclusive feeding groups on the basis of the type and amount of milk consumed: breast milk (BM), <600 mL formula milk/d (FM_low_), ≥600 mL formula milk/d (FM_high_), <600 mL cow milk/d (CM_low_), and ≥600 mL cow milk/d (CM_high_). Weight, height, and BMI were measured at 14 time points from birth to 10 y of age, and SD scores (SDSs) were calculated. Dietary energy and macronutrient intakes were available at 7 time points.

**Results: **CM_high_ children were heavier than were BM children from 8 mo to 10 y of age with weight differences (after adjustment for maternal education, smoking, and parity) ≥0.27 SDSs and an average of 0.48 SDSs. The maximum weight difference was at 18 mo of age (0.70 SDS; 95% CI: 0.41, 1.00 SDS; *P* = <0.0001). CM_high_ children were taller at some ages (25-43 mo; *P* < 0.01) and had greater BMI SDSs from ≥8 mo of age (at 9 y of age; *P* = 0.001). FM_high_ children were heavier and taller than were BM children from 8 to 37 mo of age. There were marked dietary differences between milk groups at 8 mo of age, some of which persisted to 18 mo of age. Adjustments for current energy and protein intakes did not attenuate the growth differences observed.

**Conclusions:** The feeding of high volumes of cow milk in late infancy is associated with faster weight and height gain than is BM feeding. The feeding of bottle-fed infants with high volumes of cow milk in late infancy may have a persisting effect on body habitus through childhood.

## INTRODUCTION

There is considerable ongoing debate regarding the effects of early growth and nutrition on the development of childhood obesity and its associated health risks. A rapid weight gain during infancy has been linked with obesity in early childhood (1, 2) and energy intake at 4 mo of age in formula-fed but not breastfed infants, predicting greater weight and BMI at 5 y of age (3). Elevated BMI in childhood has, in turn, been shown to be associated with increased coronary heart disease risk in adulthood (4) in addition to adverse social and economic outcomes in young adulthood (5). However, little is known about the effects of the infant diet after the introduction of complementary foods on subsequent growth (6). A Cochrane review in 2011 highlighted the need for more studies in obesity prevention to be conducted in the very young (7), and clear evidence for ways of preventing excessive weight gain in infancy and early childhood is limited. A systematic review by Owen et al. (8) concluded that, although breastfeeding results in slightly lower BMI in childhood and adulthood than formula feeding does, the difference is small and may be influenced by publication bias and confounding factors. In a subsequent meta-analysis and systematic review, rapid weight gain during the first year of life was identified as having a strong independent association with subsequent childhood overweight (9) with breastfed infants having 15% lower odds of childhood overweight than did nonbreastfed infants (9).

A large number of publications related to potential links between early feeding practices and later obesity have been questionnaire based (10–13) and may be subject to recall bias or inaccuracy (12, 14–17). A study that looked at risk of iron deficiency in the latter part of infancy, which had records of foods as eaten (18), observed that infants fed high volumes of cow milk or formula did not appear to sufficiently downregulate their energy intakes from solids. Conversely, breastfed infants appeared to adjust their energy intakes from solids depending on the volume of breast milk (BM)^6^ consumed. The aim of this study was to examine the consumption of different types of milk in the latter part of infancy and their effects on energy intake, subsequent growth, and BMI, with particular focus on the consumption of cow milk and the volume of bottle-fed milk consumed.

## METHODS

Subjects were from the Children in Focus (CIF) substudy, which included a 10% convenience sample of children taking part in the larger Avon Longitudinal Study of Parents and Children (ALSPAC) that involved >14,000 pregnant women and their subsequent offspring (19). These women were resident in a geographically defined area of South-West England and had an expected date of delivery between April 1991 and December 1992. CIF subjects, who were selected from those born in the final 6 mo of the recruitment period and who attended at least one visit (*n* = 1432) were invited to research clinics at 4, 8, and 12 mo of age and every 6th mo to the age of 61 mo and again at 7, 9, and 10 y of age. The current study was restricted to singleton children born at term (≥37 wk of gestation) with dietary information at 8 mo of age (*n* = 1112). Ethical approval for the study was obtained from the ALSPAC Law and Ethics Committee and 3 local research ethics committees. Attendance at research clinics (and the completion of questionnaires) was considered by the ethics committees as implicit consent. Assent was obtained from children [i.e., if they objected to anything, the measure was not started (or continued)].

Birth weight and length were collected as close to the birth as possible by trained ALSPAC staff. At the research clinics, weight was measured with the use of SECA scales (Seca Ltd.) to 61 mo of age and with the use of Tanita scales (Tanita Ltd.) at 7, 9, and 10 y of age. Length was measured to the age of 2 y with the use of a Kiddimetre measuring mat (Raven Equipment Ltd.), and height was measured from age 2 y onwards with the use of a Leicester height measure (Cranlea).

Dietary information was obtained when children were 8, 18, 43, and 61 mo and 7 and 10 y of age with the use of structured 3-d unweighed food records that were completed by each child’s main carer, usually the mother. The dietary method has been described in full at ages 8 mo (20), 18 mo (21), and 43 mo (22). Briefly, parents were instructed to record all foods and drinks consumed by their children in household measures including a description of any leftovers on one weekend and 2 weekdays (not necessarily consecutive). At 8, 18, and 43 mo and 10 y of age, a nutrition fieldworker checked through the diary with the parent to add additional details as necessary. Food weights and codes were allocated by the fieldworker with the aid of a coding program and combined with British food tables (23) to calculate mean daily food, drink, and nutrient intakes. BM volume was calculated according to the duration of feeds as recorded in the food records, with allowance for 10 mL/min to a maximum of 100 mL/feed (24). This method has been validated in 99 infants with the use of stable isotopes and was shown to provide reasonably reliable estimates of BM intake in the field (25).

Infants were categorized in a hierarchical manner according to the type of milk consumed at 8 mo as follows: a BM group (BM with or without some cow milk but no formula); a formula-milk group (formula with or without some BM and/or cow milk); and a cow-milk group (CM) (cow milk but no formula or BM). The amount of each type of milk consumed in each group is shown in **Supplemental Table S1**. Note that small amounts of cow milk included in the BM or formula groups were for use with complementary foods such as cereals. The cow-milk and formula groups were subdivided by the volume of milk taken per day as follows: <600 mL (<21oz) [<600 mL cow milk/d (CM_low_) and <600 mL formula milk/d (FM_low_)] and ≥600 mL (≥21oz) [≥600 mL cow milk/d (CM_high_) and ≥600 mL formula milk/d (FM_high_)] on the basis of UK infant-feeding recommendations (26, 27).

Growth data (height, weight, and BMI) was standardized against the United Kingdom 1990 growth reference (28, 29) with the use of the LMS method (30). The SD scores (SDSs) produced accounted for the sex and exact age of the child at measurement. Previous work by Ong et al. (2) developed variables showing the rate of growth of these children between birth and 2 y of age at 3 levels as follows: children showing “rapid growth” by crossing weight percentiles upwards (>0.67 SDS), children staying at the same percentile, and children showing “slow growth” by crossing percentiles downward (greater than −0.67 SDS).

A mixed-model linear regression was used to investigate the effect of each milk group on weight, length and height, and BMI SDSs to take account of the varying number of repeated measures for each child. All analyses were adjusted for maternal education, maternal smoking in pregnancy, and parity (collected via a self-completed questionnaire administered to the mother during pregnancy) because these have previously been shown to influence infant growth (31, 32). To allow for the possible changing effect of these confounders and milk groups with time, interaction terms were incorporated. The banded age at each assessment was treated as a categorical variable that allowed milk-group differences to be tracked across time without any assumption as to the nature of the trend. In addition to these fixed effects, a random effect that represented the between-child variability was included in the model. To avoid sex and age differences being confounded with differences of milk group by time, outcomes were standardized and normalized with the use of the LMS method. Secondary analyses took into account additional adjustment for current energy and protein intakes; these analyses restricted the data to the 7 time points where the dietary data were available.

Associations between milk groups and average daily energy and macronutrient intakes and milk volumes were also investigated with the use of linear regression with adjustment for sex only. The pairwise analysis included Bonferroni corrections for multiple testing. These analyses were performed cross-sectionally at each age that dietary data were collected. All analyses were performed with the use of SPSS software (version 12.0.1) (SPSS Inc.) or Stata software (version 12.0) (Statacorp LP).

## RESULTS

Dietary data were available for 1112 children at 8 mo of age (82% of the CIF subgroup) of whom 141 children (12.7%) were breastfed, 824 children (74.1%) were formula fed, and 147 children (13.2%) were fed with cow milk as their only milk drink. The numbers of children in each group who consumed either BM or high or low volumes of formula or cow milk are shown in **Table 1**. This table also shows that mothers who were feeding cow milk had lower educational attainment than did either breastfeeding or formula-feeding mothers (*n* = 1062 with all confounders).

**TABLE 1:** Associations between maternal and child characteristics and milk-feeding groups at 8 mo of age (full-term singletons with all confounders: *n* = 1062) and unadjusted associations between milk-feeding groups and outcomes at birth (maximum *n* = 1112)^1^

	Dietary group						
Characteristic	BM	FM_low_	FM_high_	CM_low_	CM_high_	Overall	*P*
Maternal							
Education, *n* (%)							0.001
Low	22 (16.1)	121 (21.1)	54 (25.5)	20 (27.8)	22 (32.8)	239 (22.5)	
Medium	38 (27.7)	205 (35.7)	73 (34.4)	28 (38.9)	30 (44.8)	374 (35.2)	
High	77 (56.2)	248 (43.2)	85 (40.1)	24 (33.3)	15 (22.4)	449 (42.3)	
Smoking, *n* (%)							0.151
No	122 (89.1)	504 (87.8)	181 (85.4)	64 (88.9)	52 (77.6)	923 (86.9)	
Yes	15 (10.9)	70 (12.2)	31 (14.6)	8 (11.1)	15 (22.4)	139 (13.1)	
Parity, *n* (%)							<0.001
0	42 (30.7)	301 (52.4)	112 (52.8)	23 (31.9)	16 (23.9)	494 (46.5)	
1	53 (38.7)	190 (33.1)	59 (27.8)	30 (41.7)	22 (32.8)	354 (33.3)	
≥2	42 (30.7)	83 (14.5)	41 (19.3)	19 (26.4)	29 (43.3)	214 (20.2)	
Child							
Sex, *n* (%)							0.045
M	68 (49.6)	299 (52.1)	133 (62.7)	38 (52.8)	41 (61.2)	579 (54.5)	
F	69 (50.4)	275 (47.9)	79 (37.3)	34 (47.2)	26 (38.8)	483 (45.5)	
Weight at birth, kg	3.51 ± 0.47^2^	3.47 ± 0.47	3.52 ± 0.49	3.61 ± 0.47	3.58 ± 0.50	3.50 ± 0.47	0.062
Length at birth, cm	50.9 ± 1.90	50.8 ± 1.91	50.9 ± 2.06	51.1 ± 1.96	51.4 ± 2.36	50.9 ± 1.97	0.284
BMI at birth, kg/m^2^	13.5 ± 1.16	13.4 ± 1.19	13.5 ± 1.26	13.8 ± 1.22	13.5 ± 1.15	13.5 ± 1.20	0.162

1 Actual *n* = 1102, 948, and 939 for weight, length, and BMI, respectively. *P* values were determined on the basis of Pearson’s chi-square test of an association for categorical variables and the *F* test (1-factor ANOVA) for continuous outcome variables. BM, breast milk; CM_high_, ≥600 mL cow milk/d; CM_low_, <600 mL cow milk/d; FM_high_, ≥600 mL formula milk/d; FM_low_ <600 mL formula milk/d.

2 Mean ± SD (all such values).

Comparisons between children in the BM group who took <6 or ≥6 breastfeeds/d at 8 mo of age showed no difference in weight, height, or BMI at any point or in energy intake at 8 mo of age (data not shown). As a result, data from BM-fed children were pooled for comparison with those of the other feeding groups.

**FIGURE 1:**
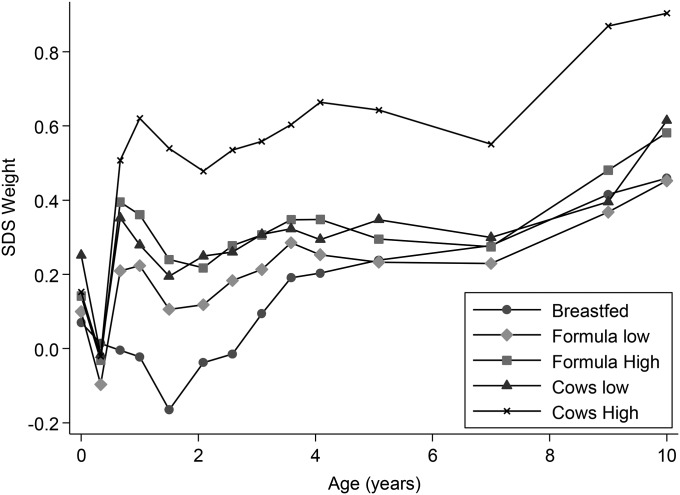
Mean weight SDSs in 5 milk-feeding groups adjusted for maternal education, smoking in pregnancy, and parity measured at 14 ages between birth and 10 y of age. Adjusted means by age are shown for the following feeding groups: breast milk (*n* = 95–136) (breastfed), <600 mL formula milk/d (*n* = 403–572) (formula low), ≥600 mL formula milk/d (*n* = 132–211) (formula high), <600 mL cow milk/d (*n* = 46–72) (cows low), and ≥600 mL cow milk/d (*n* = 42–67) (cows high). SDS, SD score.

**FIGURE 2:**
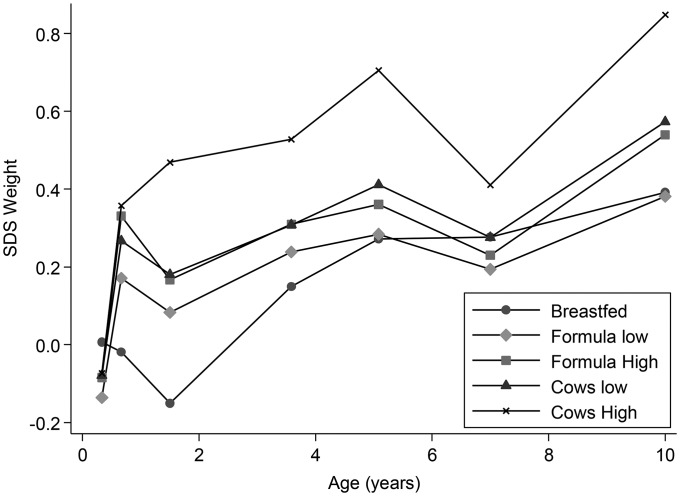
Mean weight SDSs in 5 milk-feeding groups adjusted for maternal education, smoking in pregnancy, and parity and current energy and protein intakes at each of 7 ages from 4 mo to 10 y of age. Adjusted means by age are shown for the following 5 feeding groups: breast milk (*n* = 92–136) (breastfed), <600 mL formula milk/d (*n* = 362–572) (formula low), ≥600 mL formula milk/d (*n* = 111–211) (formula high), <600 mL cow milk/d (*n* = 36–72) (cows low), and ≥600 mL cow milk/d (*n* = 37–67) (cows high). SDS, SD score.

No differences in weight SDSs between any of the feeding groups were observed at birth or 4 mo of age (**Figure 1**, **Table 2**). From ≥8 mo of age, children in some of the other milk-feeding groups were heavier than children in the BM group at various ages (Figure 1, Table 2) after adjustment for the known influences of maternal education, smoking in pregnancy, and parity. In the lower-volume feeding groups (FM_low_ and CM_low_) disparities in weight compared with BM children were small and transient (Table 2). The most-marked and persistent differences were between the CM_high_ and BM children with a maximum of a +0.70 SDS at 18 mo of age (Table 2; *P* < 0.0001) with differences persisting until 10 y of age (all *P* < 0.01 except at 7 y). Children who were fed high volumes of formula (FM_high_) were also heavier than BM children were, but the effect was less marked and less persistent (maximum SDS: +0.41 at 18 mo of age with a weight difference lasting until 37 mo of age). After additional adjustment for energy and protein intakes at the 7 available age points, these disparities in weight were maintained (**Figure 2**, **Table 3**).

**TABLE 2:** Differences between milk- (type and volume) and BM-feeding groups in weight, height, and BMI SDSs at 14 ages between birth and 10 y of age after adjustment for maternal education, smoking in pregnancy, and parity^1^

			Difference (95% CI)			
Variable and age	*n*	BM mean/group *P*^2^	FM_low_ − BM	FM_high_ – BM	CM_low_ – BM	CM_high_ – BM
Weight						
Birth	1052	0.07	0.03 (−0.16, 0.22)	0.07 (−0.14, 0.29)	0.18 (−0.10, 0.46)	0.08 (−0.21, 0.37)
4 mo	738	0.01	−0.11 (−0.31, 0.09)	−0.05 (−0.28, 0.19)	−0.03 (−0.33, 0.27)	−0.03 (−0.35, 0.28)
8 mo	1058	−0.00***	0.21 (0.03, 0.40)*	0.40 (0.18, 0.61)***	0.36 (0.07, 0.64)*	0.51 (0.22, 0.80)***
12 mo	984	−0.02***	0.25 (0.06, 0.44)*	0.38 (0.17, 0.60)***	0.30 (0.02, 0.59)*	0.64 (0.35, 0.94)***
18 mo	933	−0.17***	0.27 (0.08, 0.46)**	0.41 (0.19, 0.62)***	0.36 (0.07, 0.65)*	0.70 (0.41, 1.00)***
25 mo	904	−0.04**	0.16 (−0.04, 0.35)	0.26 (0.03, 0.48)*	0.29 (−0.01, 0.58)	0.52 (0.22, 0.81)***
31 mo	894	−0.02**	0.20 (0.01, 0.39)*	0.29 (0.07, 0.51)**	0.27 (−0.02, 0.57)	0.55 (0.25, 0.85)***
37 mo	858	0.09*	0.12 (−0.07, 0.31)	0.21 (−0.01, 0.44)	0.21 (−0.08, 0.51)	0.46 (0.16, 0.77)**
43 mo	858	0.19	0.09 (−0.10, 0.29)	0.16 (−0.07, 0.38)	0.13 (−0.16, 0.43)	0.41 (0.11, 0.72)**
49 mo	837	0.20*	0.05 (−0.15, 0.24)	0.14 (−0.08, 0.37)	0.09 (−0.21, 0.39)	0.46 (0.16, 0.77)**
61 mo	803	0.24	−0.01 (−0.20, 0.19)	0.06 (−0.17, 0.28)	0.11 (−0.19, 0.41)	0.40 (0.10, 0.71)*
7 y	780	0.28	−0.05 (−0.24, 0.15)	−0.00 (−0.23, 0.22)	0.02 (−0.28, 0.32)	0.27 (−0.04, 0.58)
9 y	740	0.42**	−0.05 (−0.25, 0.15)	0.07 (−0.16, 0.30)	−0.02 (−0.33, 0.29)	0.45 (0.14, 0.77)**
10 y	727	0.46*	−0.01 (−0.21, 0.19)	0.12 (−0.11, 0.35)	0.16 (−0.15, 0.46)	0.44 (0.13, 0.76)**
Height						
Birth	905	0.20	0.05 (−0.13, 0.23)	0.01 (−0.19, 0.22)	0.14 (−0.14, 0.42)	0.30 (0.02, 0.58)*
4 mo	736	−0.01	−0.01 (−0.20, 0.18)	0.07 (−0.15, 0.29)	0.11 (−0.18, 0.39)	0.09 (−0.21, 0.38)
8 mo	1058	0.04*	0.12 (−0.06, 0.30)	0.25 (0.04, 0.45)*	0.12 (−0.14, 0.39)	0.43 (0.15, 0.70)**
12 mo	983	0.01**	0.13 (−0.05, 0.31)	0.26 (0.05, 0.46)*	0.13 (−0.14, 0.40)	0.46 (0.18, 0.74)**
18 mo	934	−0.11***	0.17 (−0.01, 0.35)	0.39 (0.19, 0.60)***	0.27 (−0.00, 0.54)	0.60 (0.32, 0.88)***
25 mo	841	−0.17***	0.12 (−0.07, 0.30)	0.34 (0.13, 0.55)**	0.20 (−0.08, 0.47)	0.47 (0.18, 0.75)**
31 mo	858	−0.14*	0.13 (−0.05, 0.31)	0.27 (0.06, 0.48)*	0.17 (−0.10, 0.45)	0.42 (0.14, 0.71)**
37 mo	847	−0.15*	0.10 (−0.08, 0.28)	0.25 (0.04, 0.46)*	0.16 (−0.12, 0.43)	0.44 (0.15, 0.73)**
43 mo	850	−0.10*	0.11 (−0.08, 0.29)	0.22 (0.01, 0.43)*	0.02 (−0.26, 0.29)	0.41 (0.13, 0.70)**
49 mo	836	0.09	0.03 (−0.15, 0.22)	0.16 (−0.05, 0.37)	0.02 (−0.26, 0.30)	0.31 (0.02, 0.60)*
61 mo	803	0.01	−0.01 (−0.19, 0.17)	0.08 (−0.13, 0.29)	−0.02 (−0.30, 0.26)	0.25 (−0.04, 0.54)
7 y	783	0.27	−0.05 (−0.23, 0.13)	0.02 (−0.20, 0.23)	−0.10 (−0.38, 0.18)	0.16 (−0.13, 0.45)
9 y	740	0.40	−0.09 (−0.28, 0.09)	−0.00 (−0.22, 0.21)	−0.17 (−0.46, 0.11)	0.12 (−0.17, 0.42)
10 y	727	0.41	−0.05 (−0.24, 0.14)	0.07 (−0.15, 0.28)	−0.05 (−0.34, 0.23)	0.30 (0.01, 0.60)*
BMI						
Birth	896	0.24	−0.07 (−0.27, 0.12)	−0.03 (−0.25, 0.19)	0.17 (−0.12, 0.47)	−0.14 (−0.44, 0.16)
4 mo	736	−0.03	−0.16 (−0.36, 0.04)	−0.15 (−0.38, 0.09)	−0.14 (−0.44, 0.17)	−0.15 (−0.47, 0.17)
8 mo	1058	−0.03*	0.18 (−0.00, 0.37)	0.32 (0.11, 0.53)**	0.37 (0.09, 0.65)**	0.32 (0.04, 0.61)*
12 mo	982	−0.03*	0.23 (0.04, 0.41)*	0.30 (0.08, 0.51)**	0.29 (0.01, 0.58)*	0.46 (0.17, 0.75)**
18 mo	927	−0.17	0.22 (0.03, 0.41)*	0.21 (−0.01, 0.43)	0.24 (−0.05, 0.53)	0.42 (0.12, 0.72)**
25 mo	841	0.05	0.11 (−0.09, 0.30)	0.05 (−0.18, 0.27)	0.23 (−0.07, 0.53)	0.25 (−0.05, 0.55)
31 mo	857	0.09	0.13 (−0.06, 0.33)	0.15 (−0.07, 0.37)	0.23 (−0.07, 0.52)	0.35 (0.05, 0.65)*
37 mo	847	0.26	0.09 (−0.11, 0.28)	0.08 (−0.14, 0.31)	0.17 (−0.13, 0.46)	0.25 (−0.06, 0.56)
43 mo	849	0.39	0.03 (−0.17, 0.22)	0.01 (−0.21, 0.24)	0.16 (−0.14, 0.45)	0.19 (−0.11, 0.50)
49 mo	834	0.25	0.04 (−0.15, 0.24)	0.06 (−0.16, 0.29)	0.10 (−0.20, 0.40)	0.38 (0.08, 0.69)*
61 mo	798	0.32	−0.00 (−0.20, 0.19)	0.02 (−0.21, 0.24)	0.16 (−0.14, 0.46)	0.36 (0.05, 0.67)*
7 y	780	0.18	−0.04 (−0.24, 0.15)	−0.03 (−0.26, 0.19)	0.12 (−0.18, 0.43)	0.27 (−0.05, 0.58)
9 y	740	0.33**	−0.03 (−0.23, 0.17)	0.06 (−0.17, 0.29)	0.07 (−0.24, 0.38)	0.54 (0.22, 0.86)**
10 y	727	0.35*	0.01 (−0.19, 0.21)	0.13 (−0.10, 0.36)	0.26 (−0.05, 0.57)	0.43 (0.11, 0.74)**

1 BM, breast milk; CM_high_, ≥600 mL cow milk/d; CM_low_, <600 mL cow milk/d; FM_high_, ≥600 mL formula milk/d; FM_low_, <600 mL formula milk/d; SDS, SD score.

2 BM mean is the adjusted mean SDS of the breast-milk group in each age band standardized with the use of United Kingdom 1990 growth references and the LMS method (28–30). *,**,***Group *P* is an overall test of whether the BM group differed from all of the other 4 feeding groups (4 df): *P < 0.05, **P < 0.01, ***P < 0.001.

**TABLE 3:** Differences between milk- (type and volume) and BM-feeding groups in weight, height, and BMI SDS at 7 ages between 4 mo and 10 y of age after adjustment for maternal education, smoking in pregnancy, and parity and current dietary energy and protein intakes at each age^1^

			Difference (95% CI)			
Variable and age	*n*	BM mean/group *P*^2^	FM_low_ − BM	FM_high_ − BM	CM_low_ − BM	CM_high_ − BM
Weight						
4 mo	715	0.01	−0.14 (−0.35, 0.07)	−0.09 (−0.34, 0.15)	−0.09 (−0.40, 0.23)	−0.08 (−0.41, 0.25)
8 mo	1058	−0.02*	0.19 (−0.00, 0.38)	0.35 (0.13, 0.57)**	0.29 (−0.01, 0.58)	0.38 (0.06, 0.69)*
18 mo	845	−0.15**	0.23 (0.03, 0.43)*	0.32 (0.09, 0.55)**	0.33 (0.03, 0.63)*	0.62 (0.31, 0.93)***
43 mo	730	0.15	0.09 (−0.11, 0.29)	0.16 (−0.07, 0.40)	0.16 (−0.15, 0.47)	0.38 (0.06, 0.70)*
61 mo	646	0.27	0.01 (−0.20, 0.22)	0.09 (−0.16, 0.33)	0.14 (−0.19, 0.47)	0.43 (0.10, 0.76)*
7 y	659	0.28	−0.08 (−0.29, 0.13)	−0.05 (−0.29, 0.20)	−0.00 (−0.32, 0.32)	0.13 (−0.20, 0.47)
10 y	720	0.39*	−0.01 (−0.21, 0.19)	0.15 (−0.09, 0.38)	0.18 (−0.13, 0.49)	0.46 (0.13, 0.78)**
Height						
4 mo	713	−0.00	−0.01 (−0.21, 0.18)	0.08 (−0.15, 0.30)	0.11 (−0.18, 0.40)	0.10 (−0.20, 0.39)
8 mo	1058	0.03*	0.12 (−0.06, 0.29)	0.22 (0.02, 0.42)*	0.13 (−0.15, 0.40)	0.41 (0.12, 0.69)**
18 mo	846	−0.11***	0.16 (−0.02, 0.34)	0.33 (0.12, 0.54)**	0.22 (−0.06, 0.50)	0.58 (0.29, 0.86)***
43 mo	724	−0.12	0.12 (−0.07, 0.30)	0.22 (0.01, 0.44)*	0.06 (−0.23, 0.35)	0.38 (0.09, 0.68)*
61 mo	645	0.07	−0.03 (−0.22, 0.16)	0.06 (−0.16, 0.28)	−0.00 (−0.30, 0.29)	0.21 (−0.09, 0.51)
7 y	660	0.31	−0.10 (−0.29, 0.09)	−0.07 (−0.29, 0.15)	−0.15 (−0.43, 0.14)	0.05 (−0.25, 0.35)
10 y	720	0.37	−0.05 (−0.24, 0.13)	0.07 (−0.15, 0.28)	−0.07 (−0.36, 0.22)	0.30 (0.00, 0.59)*
BMI						
4 mo	713	−0.04	−0.20 (−0.42, 0.02)	−0.22 (−0.48, 0.03)	−0.22 (−0.55, 0.11)	−0.22 (−0.56, 0.12)
8 mo	1058	−0.03	0.16 (−0.04, 0.35)	0.28 (0.06, 0.51)*	0.27 (−0.03, 0.57)	0.16 (−0.16, 0.48)
18 mo	839	−0.16	0.19 (−0.02, 0.39)	0.16 (−0.08, 0.39)	0.25 (−0.06, 0.56)	0.35 (0.03, 0.66)*
43 mo	723	0.35	0.02 (−0.19, 0.23)	0.02 (−0.22, 0.26)	0.16 (−0.17, 0.48)	0.20 (−0.14, 0.53)
61 mo	641	0.29	0.06 (−0.15, 0.28)	0.09 (−0.17, 0.34)	0.20 (−0.14, 0.55)	0.45 (0.10, 0.79)*
7 y	659	0.15	−0.03 (−0.24, 0.19)	−0.02 (−0.27, 0.24)	0.13 (−0.20, 0.46)	0.15 (−0.19, 0.50)
10 y	720	0.28*	0.02 (−0.19, 0.23)	0.16 (−0.08, 0.41)	0.32 (−0.01, 0.64)	0.44 (0.10, 0.78)*

1 BM, breast milk; CM_high_, ≥600 mL cow milk/d; CM_low_, <600 mL cow milk/d; FM_high_, ≥600 mL formula milk/d; FM_low_ <600 mL formula milk/d; SDS, SD score.

2 BM mean is the adjusted mean SDS of the breast-milk group in each age band standardized with the use of United Kingdom 1990 growth references and the LMS method (28–30). *,**,***Group *P* is an overall test of whether the BM group differed from all of the other 4 feeding groups (4 df): *P < 0.05, **P < 0.01, ***P < 0.001.

The greatest and most-persistent differences in length and height SDSs were evident between CM_high_ and BM groups (Table 2). From 8 to 49 mo of age and again at 10 y of age, CM_high_ children were longer and taller, on average, than were BM children (maximum difference: 0.60 SDS at 18 mo if age; Table 2) with the scale and length of these differences being marginally reduced after adjustment for current energy and protein intakes (Table 3). FM_high_ children were also longer and taller than were BM children from 8 mo of age, but the differences were less marked and less persistent (Table 2) with adjustment for energy and protein intakes showing negligible effects (Table 3). There was no evidence to suggest that length and height differed between the low-volume feeding groups (FM_low_ and CM_low_) and the BM group (Table 2).

From 8 to 18 mo, at 49 and 61 mo, and at 9 and 10 y of age, CM_high_ children had greater mean BMI SDSs than did BM children (Table 2). The differences at 18 and 61 mo of age and 10 y of age were not abolished by adjustment for energy and protein intakes (Table 3). The other feeding groups also showed greater BMI SDSs than those of BM children from 8 mo of age, but there was no evidence of this effect continuing >18 mo of age (Table 2), and adjustment for current energy and protein intakes abolished these disparities (Table 3).

There was strong evidence that differences between feeding groups varied across time, [*P*-interaction (52 df) = 3.1 × 10^−6^ (weight), 2.4 × 10^−5^ (height), and 4.0 × 10^−4^ (BMI)]. These changing patterns were primarily restricted to the early life of infants. After 43 mo of age, there was little or no evidence for interactions (**Supplemental Table S2**). During this period, feeding-group differences persisted and primarily reflected those between CM_high_ and other groups. Typically, BM had the lowest adjusted mean of these other groups. Additional adjustments for current protein and energy intakes had little effect on adjusted means with the strongest effect for the CM_high_ group, but even for this feeding group, changes ranged from only 0.03 to 0.05 SDSs.

The proportion of children who showed rapid weight gain between birth and 2 y of age was highest in the CM_high_ group (33.9%) similar in the FM_high_ (30.7%) and FM_low_ (29.0%) groups; BM (19.7%) and CM_low_ (17.2%) groups had the lowest proportion [χ^2^ (4df) = 9.88, *P* = 0.042].

**Table 4** shows daily energy, nonmilk energy, and macronutrient intakes and total milk volumes of the feeding groups. At 8 mo of age, energy intakes were higher in the FM_high_ and CM_high_ groups than in any of the other milk groups. The most-marked disparity was between BM and CM_high_ infants with a difference in average energy intake of 739 kJ/d (95% CI: 453, 1024 kJ/d; *P* < 0.001) (Table 4). At 8 mo of age, CM_high_ infants were consuming more protein and fat than were any of the other groups (all *P* < 0.001). Protein intake of CM_high_ infants was 16.8 g (95% CI: 13.6, 19.9 g) higher than in the BM group. The FM_high_ group consumed 599 kJ energy (95% CI: 391, 807 kJ energy) and 4.5 g protein/d (95% CI: 2.2, 6.7 g protein/d) more than the BM group did (both *P* < 0.001). Nonmilk energy was lower in the FM_high_ group than FM_low_ group (*P* < 0.001) and in the CM_high_ group than in the CM_low_ group (*P* = 0.001) but did not compensate fully for the additional energy consumed from milk. These differences in macronutrient intake had largely disappeared by 18 mo of age, although children from CM_high_ and FM_high_ groups continued to consume more liquid milk than did those from the other groups (all *P* < 0.05) (Table 4). At this age, 87% of children consumed whole cow milk, and 10% of children consumed low-fat milk. By 43 mo of age, milk intakes were comparable in all groups with 29% of children consuming low-fat milk. There were no differences in macronutrient intakes between the BM group and other milk groups. There were also no differences at later ages (data not shown).

**TABLE 4:** Dietary intake of energy and macronutrient intakes assessed with the use of 3-d food records kept by parents at child ages 8, 18, and 43 mo grouped by type of milk fed at 8 mo of age^1^

	BM	FM_low_	FM_high_	CM_low_	CM_high_	Overall *P*
At 8 mo of age, *n*	141	598	226	79	68	—
Total energy, kJ	3140 (3026, 3253)^a^	3298 (3243, 3353)^a^	3738 (3648, 3828)^b^	3392 (3241, 3544)^a^	3878 (3715, 4042)^b^	<0.001
Nonmilk energy	1811 (1710, 1912)^a,b^	1919 (1870, 1968)^a^	1600 (1520, 1680)^b^	2178 (2043, 2313)^c^	1774 (1628, 1919)^a^	<0.001
Protein, g	23.1 (21.8, 24.3)^a^	26.5 (25.9, 27.1)^b^	27.6 (26.6, 28.5)^b^	33.8 (32.2, 35.5)^c^	39.8 (38.1, 41.6)^d^	<0.001
Fat, g	31.3 (29.9, 32.7)^a^	30.3 (29.6, 31.0)^a^	37.4 (36.2, 38.5)^b^	33.0 (31.1, 34.8)^a^	42.5 (40.5, 44.5)^c^	<0.001
Carbohydrate, g	99.5 (95.8, 103.1)^a^	107.5 (105.7, 109.3)^b^	117.3 (114.4, 120.2)^c^	100.0 (95.1, 104.9)^a,b^	102.1 (96.8, 107.4)^a,b^	<0.001
Total milk volume, mL	461 (437, 484)^a^	493 (482, 505)^a^	771 (753, 790)^b^	441 (410, 472)^a^	760 (726, 794)^b^	<0.001
At 18 mo of age, *n*	119	480	179	60	58	—
Energy, kJ	4615 (4451, 4779)	4504 (4423, 4586)	4731 (4597, 4866)	4561 (4331, 4792)	4706 (4471, 4941)	0.050
Protein, g	39.3 (37.5, 41.1)^a^	40.4 (39.5, 41.3)^a^	44.1 (42.6, 45.6)^b,c^	40.0 (37.4, 42.6)^a^	42.3 (39.7, 44.9)^a,c^	<0.001
Fat, g	45.2 (43.1, 47.3)	45.5 (44.5, 46.6)	48.6 (46.9, 50.3)	44.7 (41.7, 47.6)	49.6 (46.6, 52.6)	0.004
Carbohydrate, g	142.0 (136.7, 147.3)	133.4 (130.8, 137.4)	136.7 (130.6, 136.0)	139.4 (133.1, 146.9)	134.8 (127.2, 142.4)	0.048
Total milk volume, mL	379 (340, 418)^a^	401 (381, 420)^a^	496 (464, 528)^b^	402 (347, 457)^a^	552 (496, 608)^b^	<0.001
At 43 mo of age, *n*	105	410	157	49	44	—
Energy, kJ	5599 (5398, 5800)	5575 (5473, 5677)	5863 (5698, 6029)	5598 (5304, 5892)	5789 (5478, 6101)	0.047
Protein, g	45.4 (43.2, 47.6)	46.0 (44.9, 47.1)	48.5 (46.7, 50.3)	45.2 (42.0, 48.5)	45.4 (42.0, 48.8)	0.119
Fat, g	54.3 (51.7, 57.0)	55.0 (53.7, 56.3)	58.0 (55.8, 60.1)	54.8 (50.9, 58.6)	56.1 (52.1, 60.2)	0.160
Carbohydrate, g	176.5 (169.9, 183.1)	172.6 (169.2, 175.9)	181.2 (175.7, 186.6)	175.6 (166.0, 185.2)	183.7 (173.6, 193.9)	0.046
Total milk volume, mL	320 (278, 362)	330 (309, 352)	369 (335, 403)	291 (230, 352)	345 (281, 410)	0.164

1 All values are means; 95% CIs in parentheses. *P* values were determined on the basis of univariate ANOVA between-group effects (4 df) adjusted for the sex of the child. Means that do not share a common superscript letter are significantly different at *P* < 0.001. BM, breast milk; CM_high_, ≥600 mL cow milk/d; CM_low_, <600 mL cow milk/d; FM_high_, ≥600 mL formula milk/d; FM_low_, <600 mL formula milk/d.

## DISCUSSION

These results show that, compared with feeding BM, feeding high volumes of cow milk (≥600 mL/d) during the latter part of infancy is associated with increased weight and BMI that persist throughout most of childhood. Feeding high volumes of cow milk is also associated with increased length and height to 4 y of age. Furthermore, feeding high volumes of infant formula (≥ 600 mL/d) is associated with increased weight and height to 3 y of age (Table 2).

Our results concerning the associations of formula with growth are in line with the studies of Butte et al. (33) and Kramer et al. (34) who showed that differences in body composition between breastfed and formula-fed infants disappeared by 2 y of age. Other studies have shown no persisting effect of either breastfeeding or the timing of the introduction of complementary foods on body composition (17, 32). However, very few studies have looked at the type or volume of milk fed by bottle. The lack of information about cow-milk intake and its possible use instead of formula may in part account for the mixed results that have been observed in previous studies of infant feeding and growth.

Our original hypothesis was that weight gain was being stimulated by a failure to downregulate energy intake from solids when large amounts of bottle milk were being ingested. Our results indicate that this effect was the case for cow milk with average daily excess energy intake close to 740 kJ and 72% more protein being taken in the high–cow-milk–fed infants than in the BM fed infants at 8 mo of age (Table 4). Higher energy (∼600 kJ) and protein intakes (19%) were also shown in the diets of infants fed high volumes of formula milk. Intake of nonmilk energy was lower in the 2 high-volume compared with the low-volume bottle-milk groups but not enough to completely compensate for the energy from the higher milk intakes.

The mechanism by which breastfed infants tend to be leaner than are nonbreastfed infants may be related to the lower protein content of BM than of cow milk and some types of infant formula (35). However, the potential role of breastfeeding in preventing adiposity has been questioned (36). Some authors have advocated limiting protein intake from late infancy to 2 y of age in an attempt to limit early adiposity rebound (37), and higher intakes of dairy protein at 12 mo of age have been linked with a higher percentage body fat at 7 y of age (38). A European multicenter randomized controlled trial showed that feeding infant formula with a high protein content (11.7% of energy) was associated with greater weight gain in the first 2 years of life than was feeding BM (∼7.6% of energy) or a lower-protein formula (7.1% of energy), but this trial showed no effect on linear growth (39). Cow milk contains 19.8% of energy from protein, and this may account for its association with both the higher weight and height shown in our study.

Because the disparities in dietary energy and protein intakes had largely disappeared by 18 mo of age, the persisting differences in growth suggest the possibility of early programming when large volumes of cow milk are fed in late infancy. Differences in growth between the high-volume cow-milk group and BM group persisted even after correcting for current energy and protein at several ages. Ong et al. (40) showed that higher insulin-like growth factor (IGF-I) concentrations predicted a greater gain in length but a slower gain in BMI in infants to 12 mo of age. Current cow-milk and dairy-product intakes has been shown to be positively associated with IGF-I concentrations in ALSPAC children at 7 y of age (41), and elevated plasma concentrations of both growth hormone and IGF-I have been shown in prepubertal children consuming cow milk (42). Furthermore, elevated serum IGF-I concentrations have been shown in young adults who had higher rates of growth as infants (43). It is possible that cow-milk intake in late infancy may have an influence on rapid growth in infants and young children via the early stimulation of IGF-I.

In our study, 13% of infants were fed cow milk as a main drink in late infancy. The 2010 United Kingdom infant-feeding survey (44) showed 42% of infants were given some cow milk in late infancy with 4% of infants (28,000 infants/y) having cow milk as their main milk drink. The United States Infant Feeding Practices Study II (2005–2007) showed that 3.4% of infants were not having breast milk or formula by 10 mo of age (45). In the United States at 9 mo of age, 33.2% of infants were fed ≥7 oz formula 4 times/d, for a total of ≥28 oz/d (>700 mL/d) (46), which is a much higher amount than that in the current study. Our study suggests that formula feeding at this high volume may be related to rapid weight and height gains in the first 2 years of life.

We followed growth in a relatively small number of children (maximum: 1058 children at 8 mo of age, which was reduced to 727 children at 10 y of age) who were born in one area of the United Kingdom. The loss to follow up and geographical restriction suggested that the findings may not be generalizable. Another limitation was that dietary data were not available for all growth points because parents were not requested to complete food records for every clinic visit to limit participant overload. Nevertheless, we had dietary information spaced out at 7 time points during the study period with the use of a well-respected food-record method (47). Because these dietary data were collected prospectively, they were unlikely to have been affected by recall bias and weight and length-height measurements were obtained under standardized conditions. The dietary data are comparable with national data from similar aged children (48). With the use of a calculation to assess BM intake, there may have been slight differences than for actual intake; nevertheless, it is on the basis of a method that has been shown to give a reasonable estimate of BM intake (25).

Differences in early infant weight have been shown to influence later feeding practices with larger infants being introduced to solids earlier (49, 50), which may have led to growth differences. However, in our study there, was no difference in weight SDSs between milk groups at birth or 4 mo of age, which made it less likely that initial weaning practices would have differed between groups (Figure 1, Table 2).

With any observational study, there is the possibility that the results may have been biased because of residual confounding. In the models, we included the main factors relating to childhood growth that have been cited in the literature (maternal education, smoking, and parity), and hence, we anticipated that any bias would have been minor. We did not apply correction for multiple comparisons to the growth data, and hence, there is a possibility that some of the results may have been chance events. However, for the main conclusions, many of the tests conducted were part of the exploration of group differences after supporting evidence from interaction tests.

In conclusion, to our knowledge, this is the first study that has looked at growth in relation to children being fed cow milk instead of BM and at the amount of either cow milk or formula milk fed in late infancy. We have shown that the rate of growth in childhood may be influenced by both the type and volume of milk fed in infancy and suggest that details of milk intake should be measured in future research. Our findings strengthen the current American Academy of Pediatrics and United Kingdom Department of Health guidelines, which stress the need to not introduce cow milk as a main drink before 12 mo of age. Parents should be advised about the appropriate volume of milk to offer their children once complementary feeding is established.

## Footnotes

6 Abbreviations used: ALSPAC, Avon Longitudinal Study of Parents and Children; BM, breast milk; CIF, Children in Focus; CM_high_, ≥600 mL cow milk/d; CM_low_, <600 mL cow milk/d; FM_high_, ≥600 mL formula milk/d; FM_low_ <600 mL formula milk/d; IGF-I, insulin-like growth factor I; SDS, SD score.
